# Comparative anatomy of the mammalian neuromuscular junction

**DOI:** 10.1111/joa.13260

**Published:** 2020-06-23

**Authors:** Ines Boehm, Abrar Alhindi, Ana S. Leite, Chandra Logie, Alyssa Gibbs, Olivia Murray, Rizwan Farrukh, Robert Pirie, Christopher Proudfoot, Richard Clutton, Thomas M. Wishart, Ross A. Jones, Thomas H. Gillingwater

**Affiliations:** ^1^ Edinburgh Medical School: Biomedical Sciences University of Edinburgh Edinburgh UK; ^2^ Euan MacDonald Centre for Motor Neurone Disease Research University of Edinburgh Edinburgh UK; ^3^ School of Medicine UNESP‐São Paulo State University Botucatu Sao Paulo Brazil; ^4^ Faculty of Medicine Department of Anatomy King Abdulaziz University Jeddah Saudi Arabia; ^5^ The Roslin Institute and R(D)SVS University of Edinburgh Edinburgh UK

**Keywords:** comparative anatomy, mammalian, neuromuscular junction, NMJ‐morph, synapse

## Abstract

The neuromuscular junction (NMJ)—a synapse formed between lower motor neuron and skeletal muscle fibre—represents a major focus of both basic neuroscience research and clinical neuroscience research. Although the NMJ is known to play an important role in many neurodegenerative conditions affecting humans, the vast majority of anatomical and physiological data concerning the NMJ come from lower mammalian (e.g. rodent) animal models. However, recent findings have demonstrated major differences between the cellular anatomy and molecular anatomy of human and rodent NMJs. Therefore, we undertook a comparative morphometric analysis of the NMJ across several larger mammalian species in order to generate baseline inter‐species anatomical reference data for the NMJ and to identify animal models that better represent the morphology of the human NMJ in vivo. Using a standardized morphometric platform (‘NMJ‐morph’), we analysed 5,385 individual NMJs from lower/pelvic limb muscles (EDL, soleus and peronei) of 6 mammalian species (mouse, cat, dog, sheep, pig and human). There was marked heterogeneity of NMJ morphology both within and between species, with no overall relationship found between NMJ morphology and muscle fibre diameter or body size. Mice had the largest NMJs on the smallest muscle fibres; cats had the smallest NMJs on the largest muscle fibres. Of all the species examined, the sheep NMJ had the most closely matched morphology to that found in humans. Taken together, we present a series of comprehensive baseline morphometric data for the mammalian NMJ and suggest that ovine models are likely to best represent the human NMJ in health and disease.

## Graphical abstract

Boehm and colleagues provide new insights into species‐specific differences in the morphology of the mammalian neuromuscular junction.

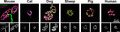

## 1 INTRODUCTION

The neuromuscular junction (NMJ) has been a focus of physiological research since the 1800s, representing an ideal, experimentally accessible, model synapse (Clarac and Pearlstein, 2007; Slater, 2008, 2015, 2017; Szule *et al*., 2015; Rudolf *et al*., 2019). More recently, it has become clear that the NMJ’s critical role in signal transmission between lower motor neuron and skeletal muscle fibre makes it a major target in many neurodegenerative and neuromuscular conditions (Murray *et al*., 2010; Rudolf *et al*., 2016; Verschuuren *et al*., 2016; Rodríguez Cruz *et al*., 2018). Whilst the physiology of the NMJ has been well‐studied across a wide range of invertebrate (Clarac and Pearlstein, 2007) and vertebrate species, including humans (Wood and Slater, 2001), far less is known about comparative NMJ morphology between mammalian species, especially with respect to humans (Jones *et al*., 2017). Moreover, small mammal animal models remain the mainstay of research into the contribution of the NMJ to many neurodegenerative conditions.

In order to better understand the form and function of the NMJ in health and disease, particularly with regard to humans, it will be important to identify animal models that more closely mimic the human condition. Given the clear differences that have been recently reported in the cellular and molecular composition of human and mouse NMJs (Jones *et al*., 2017), larger animal models might offer more appropriate alternatives. With major technological advances in gene‐editing technologies that have arisen over the past decade, there is a clear opportunity to establish large mammalian models of human disease (Eaton and Wishart, 2017). However, although significant progress has been made in understanding the morphology of lower mammalian NMJs in several species using modern imaging techniques, data from larger mammals remain sparse (Tello, 1922; Anzenbacher and Zenker, 1963; Haddix *et al*., 2018).

In the present study, we sought to establish baseline reference data sets of NMJ morphology across multiple mammalian species (mouse, cat, dog, sheep, pig, human). By utilizing our recently developed NMJ‐morph platform for comparative analysis of NMJ morphology (Jones *et al*., 2016), we have been able to generate comprehensive morphometric NMJ data from lower limb/hindlimb musculature of each species.

## 2 METHODS

### 2.1 Animals

All animal studies were performed in accordance with the Animals (Scientific Procedures) Act 1986. No animals were sacrificed specifically for this project: tissue was sampled from animals in existing studies (after experimental endpoints had been reached) or from animals submitted for euthanasia (to Dryden Farm or the Roslin Institute). Four mammalian species were investigated: cat (*N* = 3; mean age = 12.6 years), dog (*N* = 3; mean age = 6.6 years), sheep (*N* = 3; mean age = 16 months) and pig (*N* = 3; mean age = 18 months). Full details are provided in Table S1. In addition, comparative data from mouse (*N* = 3; mean age = 12 weeks) and human (*N* = 21; mean age = 67 years) were obtained from our reference archive (including previously published data; Jones *et al*., 2017). All previous human studies were covered by the requisite ethics approvals (NHS Lothian REC: 2002/1/22, 2002/R/OST/02; NHS Lothian BioResource: SR719, 15/ES/0094).

### 2.2 Tissue sampling

Animals were euthanized, and samples were harvested within 1 hr post‐mortem. Three individual animals from each species (cat, dog, sheep and pig) were sampled. To facilitate cross‐species comparison, previously studied muscles were selected (Jones *et al*., 2017): *extensor digitorum longus*,* peroneus longus*, *peroneus brevis* and *soleus*. Given the substantial difference in gross anatomy between the species (e.g. dogs lack a *soleus*; sheep and pigs have combined *peronei*), every effort was made to sample equivalent muscles based on standard descriptions of veterinary anatomy (Done *et al*., 2009; König *et al*., 2014; Aspinall and Cappello, 2015; Fails and Magee, 2018).

Full‐length muscle fibres from origin to insertion (2–3 cm in length) were dissected from each of the hindlimb muscles and immediately fixed in 4% paraformaldehyde (PFA) for 3–4 hr. Muscle samples were then washed with 1× phosphate‐buffered saline (PBS) and microdissected into small bundles of 10–15 individual fibres. All remaining fat and connective tissue were removed to reduce potential background staining.

### 2.3 NMJ immunohistochemistry

NMJs were immunolabelled by modifying an established protocol (Jones *et al*., 2017) to visualize pre‐synaptic nerve terminal proteins (SV2 and 2H3) and post‐synaptic acetylcholine receptors (AChRs).

Muscle fibres were placed in the following sequence of solutions (made up in 1×PBS unless otherwise specified): glycine (0.1 M pH 10.4) for 15 min to reduce tissue auto‐fluorescence; 1×PBS wash for 15 min; tetramethylrhodamine α‐bungarotoxin (TRITC α‐BTX, BTIU00012, VWR International Ltd.) 2 µg/ml for 15 min to label AChRs; 4% Triton X‐100 for 1.5 hr for tissue permeabilization; and then a blocking solution of 4% bovine serum albumin (BSA) and 2% Triton X‐100 for 30 min. Tissue was then incubated overnight (at room temperature) with the primary antibodies (in blocking solution): mouse monoclonal anti‐SV2 IgG (to label synaptic vesicles) and mouse monoclonal anti‐2H3 IgG (to label neurofilament) (both at 1:50 dilution; Developmental Studies Hybridoma Bank, University of Iowa). This was followed by 4 × 20 min PBS washes; overnight incubation (at 4°C) with the secondary antibodies (Alexa 488 donkey anti‐mouse IgG; 1:400 dilution; A21202, Life Technologies); and then 4 × 20 min PBS washes. Muscle samples were finally mounted on glass slides in Mowiol and kept in dark storage to prevent photobleaching.

### 2.4 Confocal imaging and NMJ‐morph analysis

NMJ images were acquired on Nikon A1R FLIM and Zeiss Axiovert LSM510 confocal microscopes using established protocols for large volume imaging (Jones *et al*., 2016, 2017). Muscle fibres were imaged on an Olympus IX71 microscope and Hamamatsu C4742‐95 camera with Openlab Improvision software using the same guidelines (Jones *et al*, 2016, 2017). For each individual muscle (*n* = 135), an average of 40–60 NMJs/muscle fibres were imaged, where possible. Muscle fibre diameters were measured subsequently from randomly identified fibres using standard light microscopy (Jones *et al*., 2016, 2017). It was not possible to record correlated NMJ and muscle fibre measurements from single identified fibres.

Image analysis was performed using the standardized ‘NMJ‐morph’ approach to quantify 21 individual morphological variables in each NMJ (including pre‐ and post‐synaptic variables and associated nerve/muscle measurements; Jones *et al*, 2016, 2017 and Boehm *et al*, 2020). In total, 5,385 NMJs were analysed across the 6 species, sampled from 135 muscles of 36 individual animals/patients (with mouse and human data pooled from Jones *et al*., 2017).

### 2.5 Statistical analysis

All statistical analyses were performed in GraphPad Prism Software (Version 8). Individual statistical tests are detailed in main text and figure legends. Statistical significance was considered to be *p* < 0.05.

## 3 RESULTS

Building on our recent work reporting marked differences between NMJ morphology in humans and mice (Jones *et al*., 2017), we initially set out to extend our knowledge of NMJ morphology across a wider range of mammalian species: cat, dog, sheep and pig. Basic background data relating to individual animals used in this study, including source and breed, are provided in Table S1.

**FIGURE 1:**
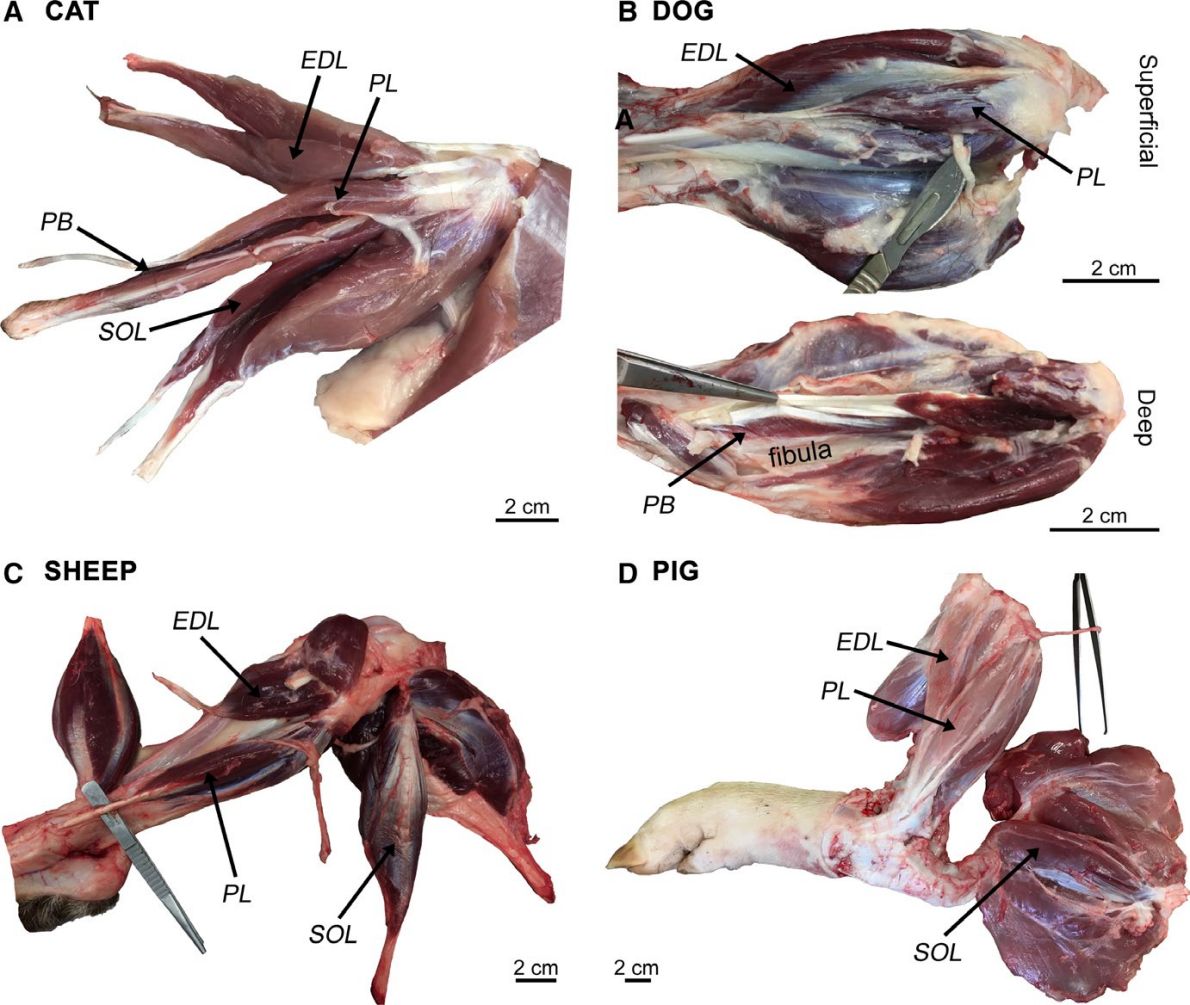
Gross anatomy of hindlimb muscles in cat, dog, sheep and pig. Representative photographs illustrating gross muscle morphology in each species. The proximal end of the limb is on the right‐hand side of the image. A block of tissue containing full‐length muscle fibres (from origin to insertion) was sampled from each of the selected muscles. Note the species‐specific absence of certain muscles—dog lacks *soleus*, and sheep and pig lack *peroneus brevis*. EDL, *Extensor Digitorum Longus*; PL, *Peroneus Longus*; PB, *Peroneus Brevis*; SOL, *Soleus*. Scale bar = 2 cm

The choice of muscles for examination (*extensor digitorum longus*,* peroneus longus*, *peroneus brevis* and *soleus*; EDL, PL, PB and S, respectively) was determined by our previously published human and mouse data sets (Jones *et al*., 2017) as well as their accessibility for dissection. The gross anatomy of hindlimb muscles in cat, dog, sheep and pig (Figure 1) does reveal some species‐specific differences (e.g. the dog lacks *soleus*, whilst both sheep and pig lack *peroneus brevis*) that likely represent functional and/or evolutionary adaptations. Nevertheless, every attempt was made to source the equivalent muscles in each species based on existing descriptions of veterinary and comparative anatomy (Done *et al*., 2009; König *et al*., 2014; Aspinall and Cappello, 2015; Fails and Magee, 2018). NMJs from each muscle/species were immunohistochemically labelled, imaged and subjected to morphometric analyses using NMJ‐morph, according to our established protocols (Jones *et al*, 2016, 2017 and Boehm *et al*, 2020). In total, 5,385 NMJs were analysed across the 6 species, sampled from 135 muscles of 36 individual animals/patients (with mouse and human reference data obtained from Jones *et al*., 2017).

**FIGURE 2:**
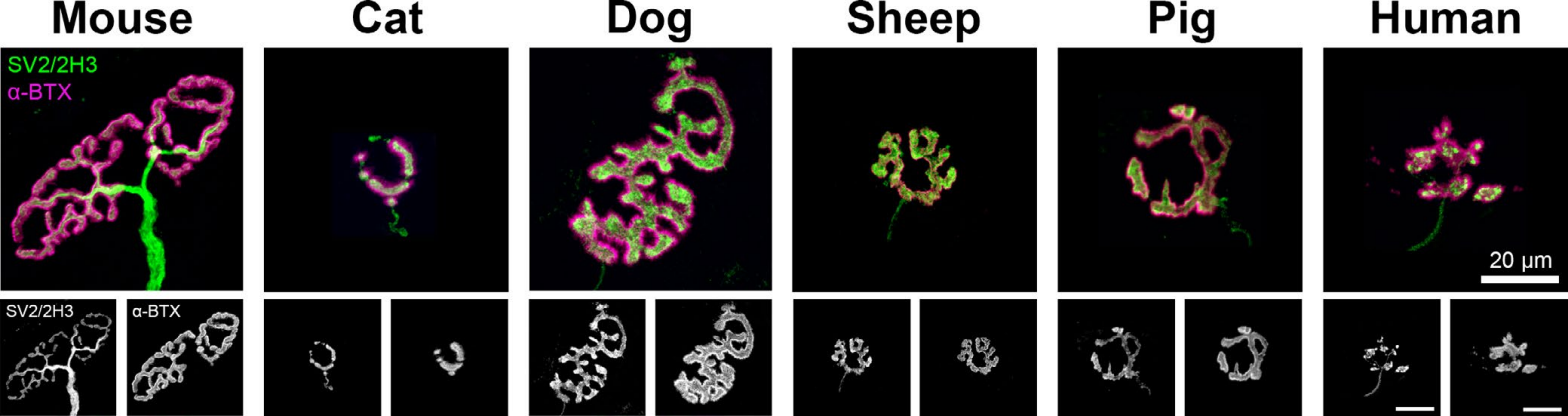
Heterogeneity of mammalian NMJs. Representative confocal micrographs of ‘average’ NMJs from each species (ranked according to body mass). The selected NMJ images most closely represent the ‘average’ morphology (size, shape) across the limb muscles sampled (EDL, soleus, peronei). Mouse NMJs are typically ‘pretzel’‐shaped; human NMJs have a ‘nummular’ morphology. Of the species represented, sheep and pig NMJs appear most similar to human NMJs. Images have been pseudo‐coloured for display purposes; all analysis has been performed on grayscale images. α‐BTX (α‐bungarotoxin), acetylcholine receptors (magenta); SV2/2H3 = synaptic vesicle and neurofilament (green). Scale bar = 20 µm (across all images)

Initial qualitative observations revealed marked inter‐species heterogeneity of NMJ morphology (Figure 2). Mouse NMJs displayed a typical ‘pretzel’‐shaped morphology, a well‐established observation in multiple previous studies (Marques *et al*, 2000). In marked contrast, human NMJs were much smaller and possessed a characteristic ‘nummular’ morphology (Jones *et al*, 2017). Of the other mammalian species, cat and dog NMJs were striking in their dissimilarity, with cat NMJs being particularly small and dog NMJs being amongst the largest examined (equivalent in size to mouse NMJs). In comparison, sheep and pig NMJs appeared quite similar to one another in overall morphology and most closely resembled the human NMJs on initial inspection. In addition, and as expected, there was notable variation of individual NMJ morphology within individual muscles (Jones *et al*, 2016, 2017).

**FIGURE 3:**
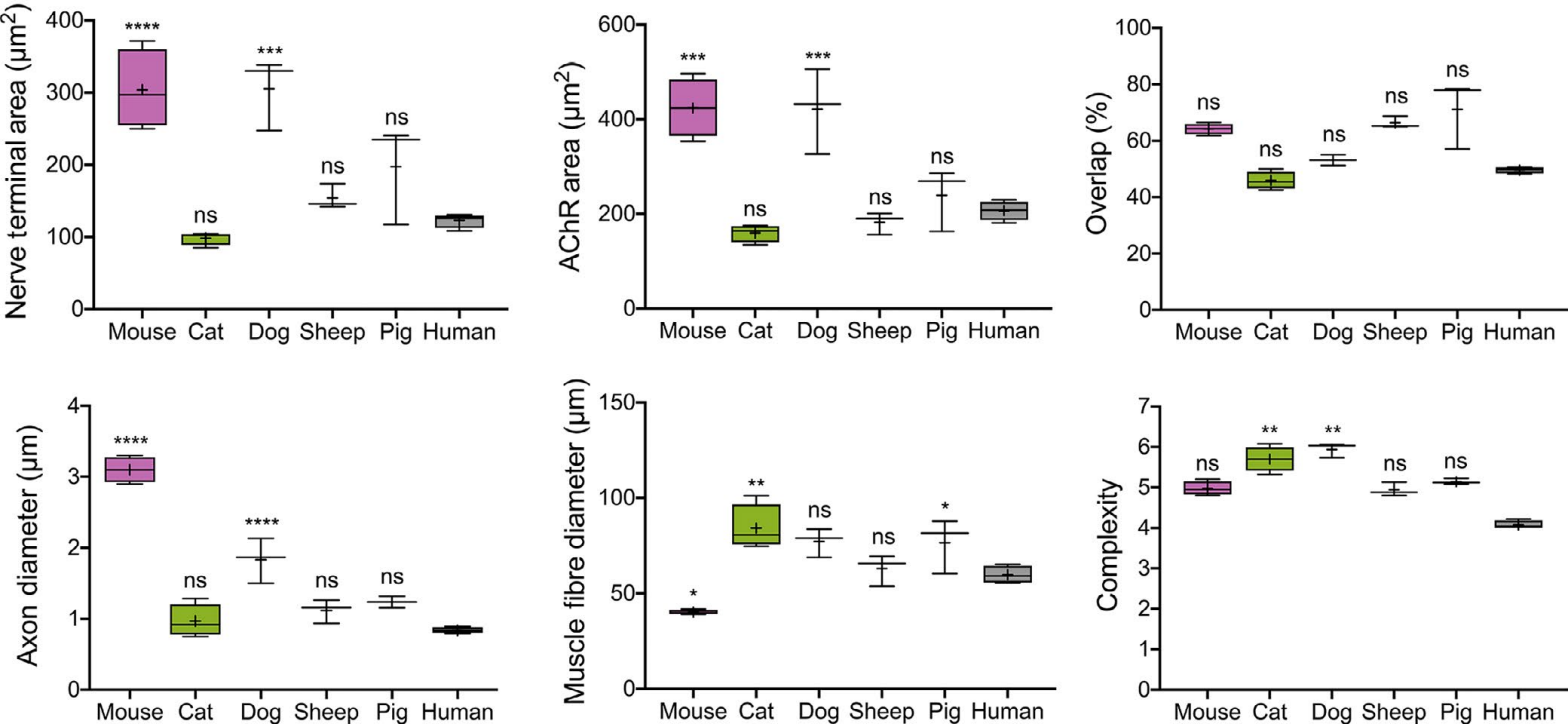
NMJ‐morph analysis reveals inter‐species variations in NMJ morphology. Comparison of ‘average’ NMJ morphology in each species for a range of pre‐ and post‐synaptic variables. Data for each morphological variable are pooled by muscle identity (EDL, PL, PB, S) for each species, and statistical comparison is made with the human NMJ. Boxes contain the mean (+) and median (line) values for each NMJ variable and extend from the 25th to 75th percentiles, with the whiskers representing the maximum and minimum values. Compared to humans, mouse and dog NMJs are significantly larger, with equivalent differences in axon calibre. In contrast, sheep and pig NMJs are the most similar to humans, with the majority of NMJ variables showing no significant differences between the species (see also Table S1). In total, 5,385 individual NMJs were analysed [cat: *N* = 3 animals, *n* = 12 muscles, 465 NMJs; dog: *N* = 3, *n* = 9, 341 NMJs; sheep: *N* = 3, *n* = 9, 313 NMJs; pig: *N* = 3, *n* = 9, 446 NMJs; mouse: *N* = 3, *n* = 24, 960 NMJs; human: *N* = 21, *n* = 72, 2860 NMJs. Mouse and human data from Jones *et al*., 2017]. One‐way ANOVA with Dunnett's post hoc analysis (for parametric variables) and Kruskal–Wallis test with Dunn's post hoc analysis (for non‐parametric variables) *p < 0.05; **p < 0.01; ***p < 0.001; ****p < 0.0001

**FIGURE 4:**
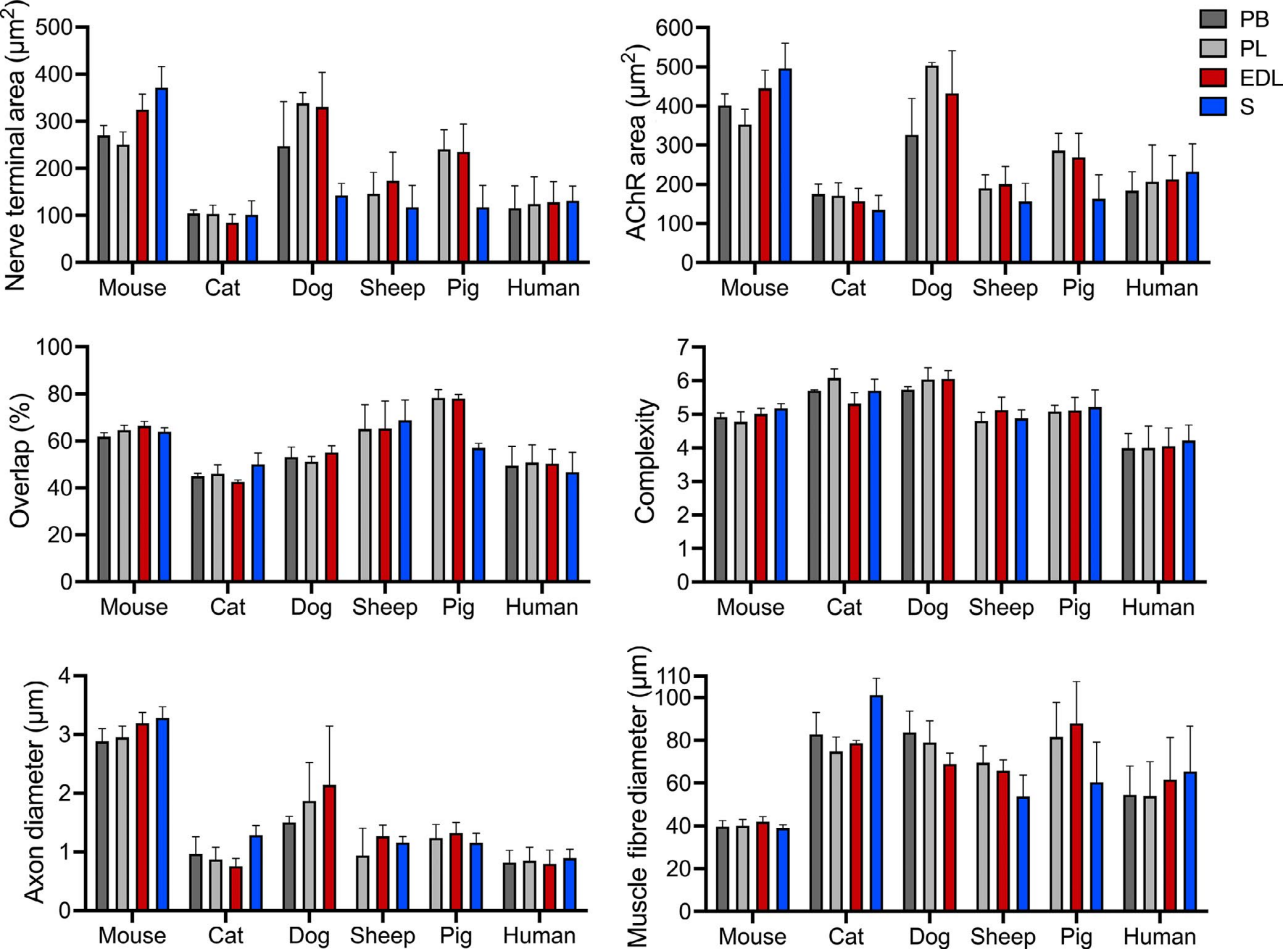
NMJ‐morph analysis of inter‐muscle variations in NMJ morphology. The pooled data in Figure 3 have been segregated to demonstrate ‘average’ NMJ morphology for individual muscles (EDL, PL, PB, S). Compared with the marked inter‐species variation, the differences between individual muscles are much less pronounced

Quantitative NMJ‐morph analysis was then performed on the complete data set of 5,385 NMJs (Figures 3 and 4; Table 1). Pooling NMJ data across all muscles sampled (EDL, PL, PB, S) facilitated a comparison of ‘average’ NMJ morphology in each species for all 21 individual pre‐ and post‐synaptic variables generated by NMJ‐morph (Table 1). Statistical comparison of these values was then performed with reference to our existing human (Jones *et al*., 2017) data set.

**TABLE 1:** Baseline morphological data for the mammalian NMJ—6 species

	Mouse *N* = 3, *n* = 24 960 NMJs	Cat *N* = 3, *n* = 12 465 NMJs	Dog *N* = 3, *n* = 9 341 NMJs	Sheep *N* = 3, *n* = 9 313 NMJs		Pig *N* = 3, *n* = 9 446 NMJs	Human *N* = 21, *n* = 72 2,860 NMJs	
Core Variables								
Pre‐synaptic								
1) Nerve Terminal Area (μm^2^)	304.0**** ± 11.7	98.5 ± 5.4	305.1*** ± 24.9		153.9 ± 14.3	197.6 ± 24.7		122.7 ± 5.98
2) Nerve Terminal Perimeter (μm)	327.4**** ± 9.4	272.1*** ± 13.9	441.0**** ± 31.7		231.7* ± 12.3	234.7* ± 18.6		151.1 ± 6.53
3) Number of Terminal Branches	30.0 ± 1.2	101.4**** ± 7.4	95.6**** ± 8.5		45.5 ± 4.4	40.0 ± 6.0		28.0 ± 1.40
4) Number of Branch Points	26.0 ± 0.9	57.6*** ± 6.9	62.9**** ± 3.5		26.4 ± 2.6	38.5* ± 3.2		14.0 ± 0.85
5) Total Length of Branches (μm)	166.7**** ± 4.8	128.7** ± 7.7	215.7**** ± 13.5		109.5 ± 8.1	134.5** ± 12.0		69.3 ± 3.28
Post‐synaptic								
6) AChR Area (μm^2^)	424.2*** ± 14.1	159.6 ± 9.3	420.8*** ± 35.1		182.2 ± 14.0	239.5 ± 25.2		206.7 ± 8.0
7) AChR Perimeter (μm)	275.8** ± 8.8	332.7**** ± 16.5	536.5**** ± 41.7		241.6* ± 15.2	221.7 ± 18.6		152.2 ± 5.8
8) Endplate Area (μm^2^)	678.2*** ± 24.3	333.2 ± 19.9	858.9**** ± 85.0		365.4 ± 16.8	384.2 ± 38.5		351.5 ± 14.4
9) Endplate Perimeter (μm)	118.8 ± 2.9	91.4 ± 3.0	139.1** ± 10.2		85.1 ± 3.8	95.5 ± 7.3		98.7 ± 2.6
10) Endplate Diameter (μm)	42.2 ± 1.2	30.9 ± 1.1	48.1** ± 3.4		30.3 ± 1.4	32.2 ± 2.0		36.0 ± 0.9
11) Number of AChR Clusters	2.6 ± 0.2	3.2 ± 0.4	3.8 ± 0.5		4.9 ± 0.4	3.7 ± 0.5		3.9 ± 0.2
Derived Variables								
Pre‐synaptic								
12) Average Length of Branches (μm)	6.7**** ± 0.2	1.4 ± 0.1	2.6 ± 0.2	3.0 ± 0.4		4.4 ± 0.6	3.0 ± 0.1	
13) Complexity	4.9 ± 0.1	5.7** ± 0.1	5.9** ± 0.1	4.9 ± 0.1		5.1 ± 0.1	4.1 ± 0.1	
Post‐synaptic								
14) Average Area of AChR Clusters (μm^2^)	238.5**** ± 8.5	75.8 ± 8.6	175.4**** ± 12.6	54.8 ± 6.8		113 ± 12.9	71.7 ± 2.7	
15) Fragmentation	0.40 ± 0.0	0.51 ± 0.0	0.53 ± 0.1	0.67 ± 0.0		0.48 ± 0.0	0.58 ± 0.0	
16) Compactness (%)	64.4 ± 0.6	49.5 ± 1.1	50.4 ± 1.8	51.0 ± 3.1		64.5 ± 2.3	61.6 ± 0.6	
17) Overlap (%)	64.2 ± 0.5	45.9 ± 1.1	53.1 ± 1.1	66.4 ± 3.0		71.1 ± 3.6	49.6 ± 1.1	
18) Area of Synaptic Contact (μm^2^)	267.9**** ± 9.5	72.4 ± 4.5	224.4** ± 18.1	122.0 ± 13.4		175.0 ± 24.2	105.2 ± 5.1	
Associated Nerve and Muscle Variables								
19) Axon Diameter (μm)	3.1**** ± 0.1	0.96 ± 0.1	1.8**** ± 0.22		1.1 ± 0.09	1.2 ± 0.1	0.84 ± 0.0	
20) Muscle Fibre Diameter (μm)	40.2* ± 0.5	84.3** ± 3.5	77.2 ± 3.33		63.0 ± 3.29	76.6 ± 6.7	59.9 ± 2.1	
21) Number of Axonal Inputs	1.0 ± 0.0	1.0 ± 0.0	1.0 ± 0.00		1.0 ± 0.00	1.1 ± 0.1	1.0 ± 0.0	

Complete NMJ‐morph data for the 6 species. Values are the mean (±*SEM*) for each NMJ variable pooled across all muscles sampled (EDL, PL, PB, S) in each species. *N*, individual animals; *n*, individual muscles; total NMJs per species listed below. All statistical comparisons performed in relation to the human data; one‐way ANOVA with Dunnett's post hoc analysis (parametric variables) and Kruskal–Wallis test with Dunn's post hoc analysis (non‐parametric variables).

No asterisk indicates non‐significant result. Mouse and human data reproduced from Jones *et al*, 2017.

* *p* < 0.05;

** *p* < 0.01;

*** *p* < 0.001;

**** *p* < 0.0001.

The majority of core NMJ variables were significantly larger in both mouse (6/11 variables) and dog (10/11 variables) compared to humans; these differences were also matched by significantly greater axonal diameters in both species. At the opposite end of the spectrum, cat NMJs were significantly smaller than human NMJs (in 5/11 core variables). In contrast, quantitative analysis of both sheep and pig NMJs revealed a closer similarity to human NMJs, with the vast majority of variables in both species (19/21 in sheep; 18/21 in pig) showing no statistically significant differences.

To determine whether these observations were reflective of NMJ morphology at the level of individual muscles, data sets from each species were segregated into distinct muscle groups: EDL, PL, PB and S (Figure 4). Compared with the marked variation in average NMJ morphology between species (Figures 2 and 3; Table 1), the differences that exist between individual muscles within a species (Jones *et al*., 2016) are much less pronounced. Moreover, there was no overt pattern to suggest a relationship between NMJ morphology and muscle fibre type (e.g. fast‐twitch/slow‐twitch) or anatomical ‘identity’ at the whole muscle level. For example, in both humans and mice, the *largest* relative NMJs were found in soleus (an archetypal slow‐twitch muscle), whereas in sheep and pigs, soleus contained the *smallest* relative NMJs (Figure 4).

**FIGURE 5:**
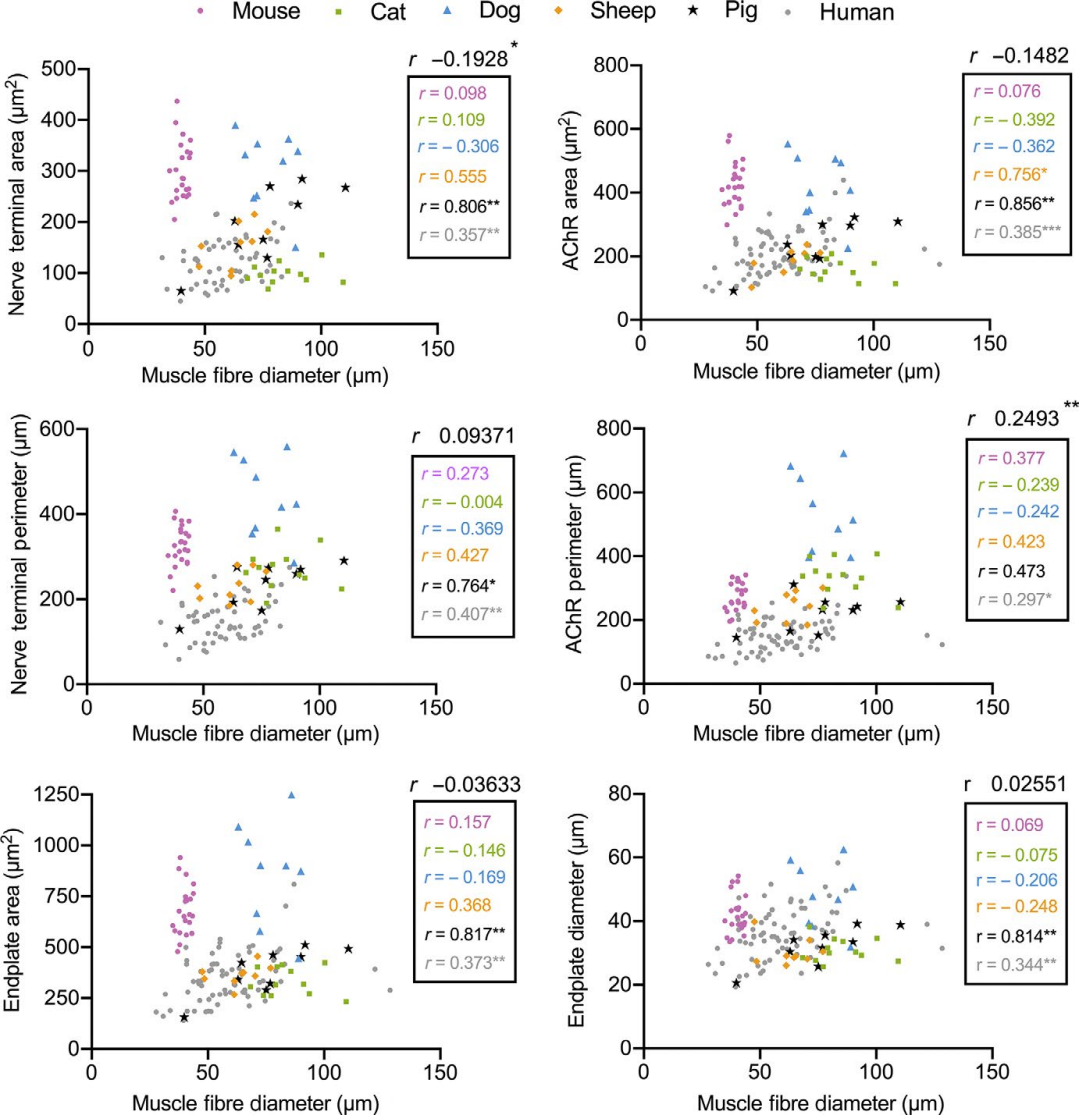
No significant relationship between NMJ morphology and muscle fibre diameter. Scatterplots demonstrating the correlation between size‐related NMJ variables and muscle fibre diameter. Each data point represents an individual muscle (mean of 40 NMJs/40 muscle fibres). Despite modest correlations for some variables, there is no significant relationship between NMJ size and muscle fibre diameter across the species investigated. Mouse = purple circles; cat = green squares; dog = blue triangles; sheep = orange diamonds; pig = black stars; Human = grey circles. Correlation coefficients (Pearson) are for individual species (within the box) and all species pooled (above the box). *p < 0.05; **p < 0.01; ***p < 0.001

We next investigated the relationship between NMJ size and muscle fibre diameter. Previous studies have suggested a significant positive correlation between NMJ size and muscle fibre diameter (Kuno *et al*., 1971; Harris and Ribchester, 1979; Slack *et al*., 1983; Balice‐Gordon and Lichtman, 1990), as well as an inverse relationship between NMJ size and body mass (Paul, 2001; Slater, 2017). We performed a correlation analysis of muscle fibre diameter against each of 18 pre‐ and post‐synaptic NMJ variables across all species (Figure 5). Despite modest correlations for individual variables in some species (e.g. pig and human; Figure 5), no overall relationship was observed for the majority of NMJ variables across the pooled data for all species. This finding suggests that muscle fibre diameter is not the sole/major determinant of NMJ size and morphology.

**FIGURE 6:**
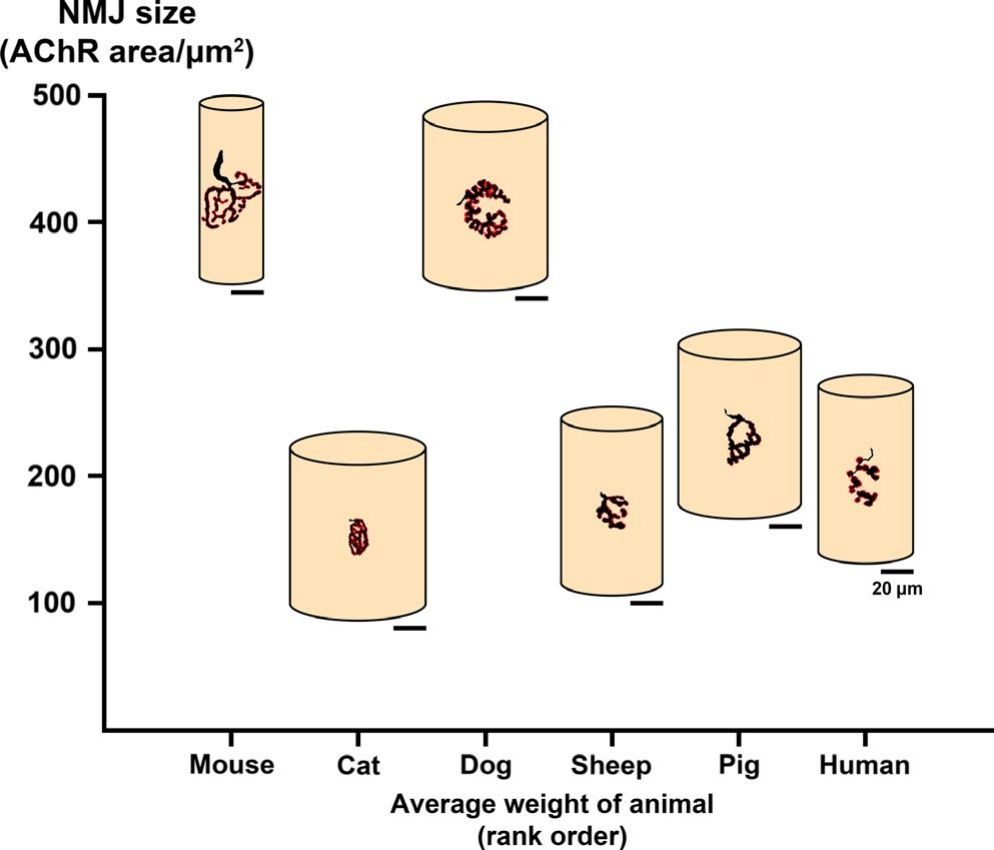
Schematic overview of NMJ morphology, muscle fibre diameter and body size. Schematic diagram illustrating the relationship between NMJ size, muscle fibre diameter and body weight. For each species, the mean values for AChR area and muscle fibre diameter are depicted (Table 1) to provide an accurate visual representation of inter‐species differences/similarities. Of the species investigated, the starkest contrast is between the two smallest animals; the mouse has the largest NMJs on the smallest muscle fibres, and the cat has the smallest NMJs on the largest muscle fibres. Sheep and pig are most similar to human, with sheep and human bearing the closest resemblance. Overall, there is no relationship between NMJ size, muscle fibre diameter and body mass; the ratio between NMJ size and muscle fibre diameter is therefore unique to each species. Scale bar = 20 µm

Finally, we wanted to determine whether relative body size/mass was contributing to NMJ size/morphology. When taken together with our previous observations, we found no clear relationship between NMJ size/morphology, muscle fibre diameter and/or body size (Figure 6). For example, the greatest disparity between NMJ size and muscle fibre diameter was observed between the two smallest animals included in the study, with the mouse possessing the largest NMJs on the smallest muscle fibres and the cat displaying the smallest NMJs on the largest muscle fibres. In contrast, the three largest species by weight (sheep, pig and human) had the most closely matched ‘NMJ size/fibre size’ pairings, with sheep and humans showing the most similarities.

## 4 DISCUSSION

The current study presents comparative reference/baseline data for NMJ morphology across 6 mammalian species (Table 1; Figure 6). We reveal marked species‐specific differences in NMJ morphology across a range of anatomically defined muscle groups in the mammalian lower limb/hindlimb. We report marked heterogeneity of NMJ morphology both within and between species, with no relationship found between NMJ morphology and muscle fibre diameter or body size. Of all the species examined in this study, the sheep NMJ was found to most closely resemble the human NMJ.

The finding of marked heterogeneity in NMJ morphology across a range of mammalian species questions the extent to which NMJ form and function in one species can automatically be translated to our understanding of NMJ form and function in another mammalian species. Whilst there are undoubtedly many aspects of NMJ form and function that have been successfully replicated across mammalian species, it is possible to envisage a scenario whereby responses to degenerative or disease stimuli could elicit distinct species‐specific responses at the NMJ. Indeed, this may (at least in part) help to explain why many studies of NMJ‐related diseases in mouse models have not been successfully translated to human patients (e.g. in motor neuron diseases such as amyotrophic lateral sclerosis; Dupuis and Loeffler, 2009; Clerc *et al*., 2016). However, this may reflect only one aspect of multi‐factorial issues contributing to the poor therapeutic translation of clinical trials (Tosolini and Sleigh 2017; Mitsumoto *et al*., 2014).

Our finding that pig, and particularly sheep, NMJs more closely resemble human NMJs than those in mice, cats and dogs suggests that the utilization of large animal models may be more appropriate and accurate for understanding human NMJs in health and disease. Indeed, many new spontaneous and genetically altered large animal models of neurological and neuromuscular disease have recently been reported (Eaton and Wishart, 2017). For example, porcine models of spinal muscular atrophy (Walters and Prather, 1969; Lorson *et al*., 2011; Prather *et al*., 2013; Duque *et al*., 2015) and ovine models of Batten disease (Weber and Pearce, 2013; Eaton *et al*., 2019) are now both available for basic, pre‐clinical and clinical studies. Thus, the incorporation of large mammalian models into research programmes is likely to yield important new insights into NMJ biology, as well as the development of effective therapies for neuromuscular conditions.

Correlation analyses of NMJ morphology and muscle fibre diameter in the present study add significant experimental support to refute the hypothesis that the former is largely dictated by the latter (Kuno *et al*., 1971; Harris and Ribchester, 1979; Slack *et al*., 1983; Balice‐Gordon and Lichtman, 1990). Our data therefore support previous studies suggesting a disconnection of NMJ morphology and muscle fibre size in rodents, primates and humans (Anzenbacher and Zenker, 1963; Jones *et al*., 2017). However, it is important to note that this observation is distinct from the relationship that has been shown to exist between the size of an NMJ and its corresponding muscle fibre when undergoing atrophy and hypertrophy in situations of muscle wasting, exercise or hormonal manipulation (e.g. as has been reported in the mouse bulbocavernosus muscle; Balice‐Gordon and Lichtman, 1990). It remains unclear, therefore, as to which factors directly determine NMJ morphology in vivo, although recent studies have suggested that the identity of the motor neuron itself is likely to exert a strong influence (Jones *et al*., 2016, 2017).

Given that muscle fibre diameter does not determine the *absolute* size/morphology of any given NMJ, it appears that the ‘ratio’ between NMJ size and muscle fibre diameter represents a unique characteristic in each species and one that is furthermore independent of body mass (Figure 6). This notion was previously proposed for different muscles within the rat (Oda, 1985), but here we extend these findings to show that it persists across multiple mammalian species.

## CONFLICT OF INTEREST

The authors declare no conflicts of interest.

## AUTHOR CONTRIBUTIONS

IB, RAJ and THG conceived and designed the study. IB, AA, ASL, AG, OM, RF, RAJ and THG performed experiments and analysed data. CL, RP, CP, RC and TMW provided access to, and guidance with, tissue sampling. AB, AA, ASL, RAJ and THG wrote the manuscript. All authors edited and approved the manuscript.

## Supporting information

Table S1

## Data Availability

All experimental data are contained within the figures and tables. All raw data files (including confocal micrographs and data spreadsheets) are freely available upon request.
