# Supplementary material for: Comparative anatomy of the mammalian neuromuscular junction
Source: J Anat. 2020 Jun 23;237(5):827–36. doi: 10.1111/joa.13260 (PMC7542190; doi:10.1111/joa.13260)
Supplement: Supplementary file 1 — Table S1 [file JOA-237-827-s001.docx]

**Boehm et al., Supplementary Table 1**

| **Species** | **Mouse** | **Cat** | **Dog** | **Sheep** | **Pig** |
| --- | --- | --- | --- | --- | --- |
| **Sex (M:F)** | 0:3 | 0:3 | 2:1 | 3:0 | 0:3 |
| **Age** | 12 weeks | 12.6 ± 3.1 years (min 12, max 16) | 6.6 ± 4.6 years (min 4, max 12) | 1.3 ± 0.2 years  (min 1.3, max 1.5) | 0.34 years  (min 0.33, max 0.35) |
| **Weight** | ≃ 19 g | 4.3 ± 1.4 kg (min 3.2, max 5.3) | ≃ 39 kg | 75 kg | 75.6 ± 7.5 kg  (min 67, max 80) |
| **Breed** | CD1 | Domestic  short-haired | Labrador,  Staffie X, Lurcher | Texel crosses | Largewhite X landrace |
| **Sampled**  **muscles** | EDL, PL, PB, S | EDL, PL, PB, S | EDL, PL, PB | EDL, PL, S | EDL, PL, S |
| **Reason for cull** | Experimental | Aggression, hyperthyroidism, chronic kidney disease | Aggression, neoplasia, joint pain | Colony management | Colony management |

**Supplementary Table 1. Background data of study animals**

All species sourced from the animal facilities at the University of Edinburgh (Centre for Discovery Brain Sciences; Roslin Institute; Dryden Farm). Ages for each animal/species equivalent to adulthood. Numerical data are mean ± standard deviation (SD). EDL: *Extensor Digitorum Longus*. PL: *Peroneus Longus*. PB: *Peroneus Brevis*. S: *Soleus*
